# 4,4′,6,6′-Tetra­bromo-2,2′-[(*E*,*E*)-ethane-1,2-diylbis(nitrilo­methanylyl­idene)]di­phenol

**DOI:** 10.1107/S1600536812029832

**Published:** 2012-07-07

**Authors:** Hadi Kargar, Reza Kia, Amir Adabi Ardakani, Muhammad Nawaz Tahir

**Affiliations:** a Department of Chemistry, Payame Noor University, PO Box 19395-3697 Tehran, I. R. of IRAN; b Department of Chemistry, Science and Research Branch, Islamic Azad University, Tehran, Iran; c Department of Physics, University of Sargodha, Punjab, Pakistan

## Abstract

The asymmetric unit of the title compound, C_16_H_12_Br_4_N_2_O_2_, comprises half of a potential tetra­dentate Schiff base ligand. The whole mol­ecule is generated by an inversion center located in the middle of the C—C bond of the ethyl­ene segment. There are intra­molecular O—H⋯N hydrogen bonds making *S*(6) ring motifs. In the crystal, no significant inter­molecular inter­actions are observed.

## Related literature

For standard values of bond lengths, see: Allen *et al.* (1987 ▶). For details of hydrogen-bond motifs, see: Bernstein *et al.* (1995 ▶). For the crystal structure of a similar compound, see: Kia *et al.* (2012 ▶).
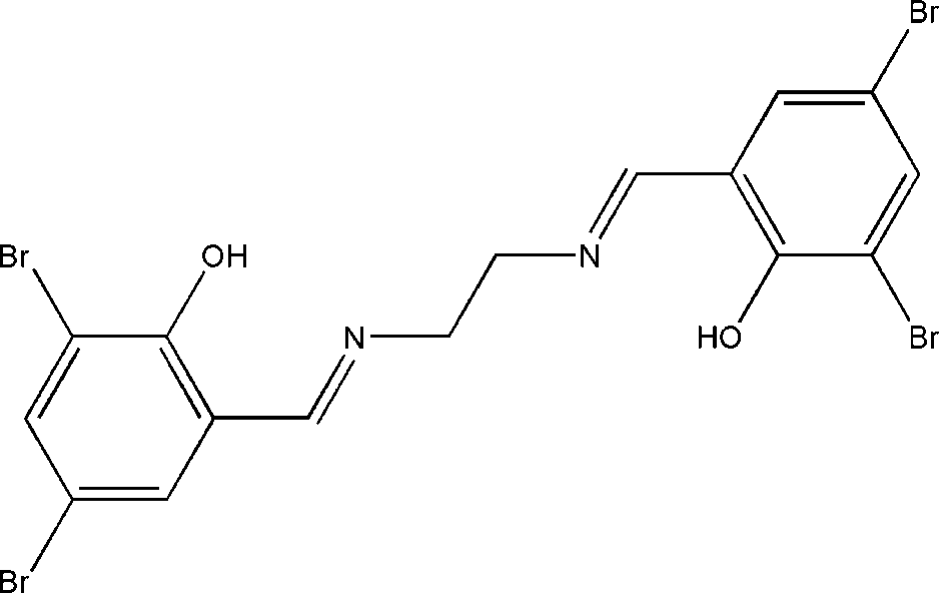


## Experimental

### 

#### Crystal data

C_16_H_12_Br_4_N_2_O_2_
*M* *_r_* = 583.92
Monoclinic, 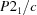

*a* = 12.723 (3) Å
*b* = 10.291 (2) Å
*c* = 6.9428 (18) Å
β = 97.046 (15)°
*V* = 902.2 (4) Å^3^
*Z* = 2
Mo *K*α radiation
μ = 8.93 mm^−1^
*T* = 291 K
0.21 × 0.14 × 0.08 mm

#### Data collection

Bruker SMART APEXII CCD area-detector diffractometer
Absorption correction: multi-scan (*SADABS*; Bruker, 2005 ▶) *T*_min_ = 0.256, *T*_max_ = 0.535
6730 measured reflections
1981 independent reflections
1086 reflections with *I* > 2σ(*I*)
*R*_int_ = 0.075

#### Refinement

*R*[*F* ^2^ > 2σ(*F* ^2^)] = 0.069
*wR*(*F* ^2^) = 0.202
*S* = 0.98
1981 reflections
109 parameters
H-atom parameters constrained
Δρ_max_ = 1.40 e Å^−3^
Δρ_min_ = −0.90 e Å^−3^

### 

Data collection: *APEX2* (Bruker, 2005 ▶); cell refinement: *SAINT* (Bruker, 2005 ▶); data reduction: *SAINT*; program(s) used to solve structure: *SHELXS97* (Sheldrick, 2008 ▶); program(s) used to refine structure: *SHELXL97* (Sheldrick, 2008 ▶); molecular graphics: *SHELXTL* (Sheldrick, 2008 ▶); software used to prepare material for publication: *SHELXTL* and *PLATON* (Spek, 2009 ▶).

## Supplementary Material

Crystal structure: contains datablock(s) global, I. DOI: 10.1107/S1600536812029832/su2470sup1.cif

Structure factors: contains datablock(s) I. DOI: 10.1107/S1600536812029832/su2470Isup2.hkl

Additional supplementary materials:  crystallographic information; 3D view; checkCIF report

## Figures and Tables

**Table 1:** Hydrogen-bond geometry (Å, °)

*D*—H⋯*A*	*D*—H	H⋯*A*	*D*⋯*A*	*D*—H⋯*A*
O1—H1⋯N1	0.82	1.83	2.573 (7)	151

**Fig. 1.**
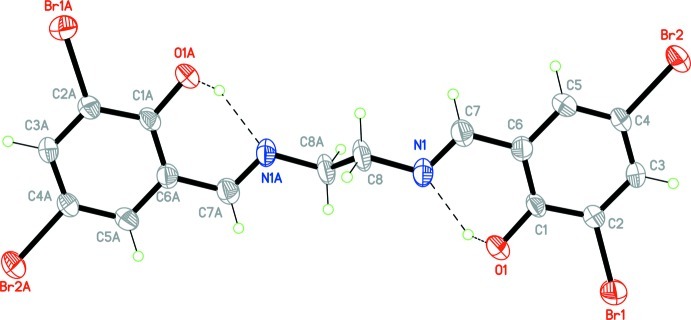
The molecular structure of the title compound, showing 40% probability displacement ellipsoids and the atomic numbering [symmetry code for suffix A: -x + 1, -y, -z + 1]. The intramolecular O-H···N hydrogen bonds are shown as dashed lines.

## supplementary crystallographic information

### Comment

In continuation of our work on the crystal structure analyses of Schiff base ligands (Kargar *et al.*, (2011); Kia *et al.*, (2010), we synthesized the title compound and report herein on its crystal structure.

The asymmetric unit of the title compound, Fig. 1, comprises half of a potentially tetradentate Schiff base ligand. The molecule is located about an inversion center, located in the middle of the C8—C8A bond of the ethylene segment. The bond lengths (Allen *et al.*, 1987) and angles are within the normal ranges and are comparable to those reported for a similar compound (Kia *et al.*, 2012). The intramolecular O—H···N hydrogen bonds (Table 1) make *S(6)* ring motifs (Bernstein *et al.*, 1995).

In the crystal, there are no significant intermolecular interactions present.

### Experimental

The title compound was synthesized by adding 3,5-dibromosalicylaldehyde (2 mmol) to a solution of ethylenediamine (1 mmol) in ethanol (30 ml). The mixture was refluxed with stirring for 30 min. The resultant solution was filtered. Yellow single crystals of the title compound suitable for *X*-ray structure determination were recrystallized from ethanol by slow evaporation of the solvents at room temperature over several days.

### Refinement

The O-bound H atom was located in a difference Fourier map and constrained to refine on the parent O atom with U_iso_(H) = 1.5U_eq_(O). The C-bound H-atoms were included in calculated positions and treated as riding atoms: C—H = 0.93 and 0.97 Å for CH and CH_2_ H-atoms, respectively, with U_iso_(H) = 1.2U_eq_(C).

### Crystal data

**Table:**

C_16_H_12_Br_4_N_2_O_2_	*F*(000) = 556
*M**_r_* = 583.92	*D*_x_ = 2.150 Mg m^−^^3^
Monoclinic, *P*2_1_/*c*	Mo *K*α radiation, λ = 0.71073 Å
Hall symbol: -P 2ybc	Cell parameters from 1563 reflections
*a* = 12.723 (3) Å	θ = 2.5–27.4°
*b* = 10.291 (2) Å	µ = 8.93 mm^−^^1^
*c* = 6.9428 (18) Å	*T* = 291 K
β = 97.046 (15)°	Block, pale-yellow
*V* = 902.2 (4) Å^3^	0.21 × 0.14 × 0.08 mm
*Z* = 2	

### Data collection

**Table:**

Bruker SMART APEXII CCD area-detector diffractometer	1981 independent reflections
Radiation source: fine-focus sealed tube	1086 reflections with *I* > 2σ(*I*)
Graphite monochromator	*R*_int_ = 0.075
φ and ω scans	θ_max_ = 27.1°, θ_min_ = 1.6°
Absorption correction: multi-scan (*SADABS*; Bruker, 2005)	*h* = −16→13
*T*_min_ = 0.256, *T*_max_ = 0.535	*k* = −13→12
6730 measured reflections	*l* = −8→8

### Refinement

**Table:**

Refinement on *F*^2^	Primary atom site location: structure-invariant direct methods
Least-squares matrix: full	Secondary atom site location: difference Fourier map
*R*[*F*^2^ > 2σ(*F*^2^)] = 0.069	Hydrogen site location: inferred from neighbouring sites
*wR*(*F*^2^) = 0.202	H-atom parameters constrained
*S* = 0.98	*w* = 1/[σ^2^(*F*_o_^2^) + (0.1116*P*)^2^] where *P* = (*F*_o_^2^ + 2*F*_c_^2^)/3
1981 reflections	(Δ/σ)_max_ < 0.001
109 parameters	Δρ_max_ = 1.40 e Å^−^^3^
0 restraints	Δρ_min_ = −0.90 e Å^−^^3^

### Special details

**Table:**

Geometry. All esds (except the esd in the dihedral angle between two l.s. planes) are estimated using the full covariance matrix. The cell esds are taken into account individually in the estimation of esds in distances, angles and torsion angles; correlations between esds in cell parameters are only used when they are defined by crystal symmetry. An approximate (isotropic) treatment of cell esds is used for estimating esds involving l.s. planes.
Refinement. Refinement of F^2^ against ALL reflections. The weighted R-factor wR and goodness of fit S are based on F^2^, conventional R-factors R are based on F, with F set to zero for negative F^2^. The threshold expression of F^2^ > 2sigma(F^2^) is used only for calculating R-factors(gt) etc. and is not relevant to the choice of reflections for refinement. R-factors based on F^2^ are statistically about twice as large as those based on F, and R- factors based on ALL data will be even larger.

### Fractional atomic coordinates and isotropic or equivalent isotropic displacement parameters (Å^2^)

**Table:**

	*x*	*y*	*z*	*U*_iso_*/*U*_eq_	
Br1	0.30804 (8)	0.64680 (8)	0.49803 (17)	0.0684 (4)	
Br2	−0.05455 (7)	0.36429 (9)	0.18237 (15)	0.0615 (4)	
O1	0.4014 (4)	0.3794 (5)	0.5301 (8)	0.0514 (14)	
H1	0.4198	0.3042	0.5527	0.077*	
N1	0.3995 (5)	0.1295 (5)	0.5201 (10)	0.0456 (17)	
C1	0.3005 (5)	0.3720 (7)	0.4544 (11)	0.0373 (17)	
C2	0.2407 (6)	0.4862 (7)	0.4249 (11)	0.0408 (18)	
C3	0.1366 (6)	0.4860 (7)	0.3445 (11)	0.0443 (19)	
H3A	0.0991	0.5633	0.3236	0.053*	
C4	0.0901 (6)	0.3697 (7)	0.2964 (12)	0.0417 (18)	
C5	0.1429 (5)	0.2529 (7)	0.3262 (10)	0.0399 (18)	
H5A	0.1087	0.1748	0.2926	0.048*	
C6	0.2490 (6)	0.2542 (7)	0.4080 (11)	0.0398 (18)	
C7	0.3031 (6)	0.1305 (8)	0.4442 (11)	0.044 (2)	
H7A	0.2677	0.0529	0.4124	0.053*	
C8	0.4522 (6)	0.0026 (8)	0.5551 (13)	0.053 (2)	
H8A	0.4034	−0.0668	0.5118	0.063*	
H8B	0.4743	−0.0087	0.6928	0.063*	

### Atomic displacement parameters (Å^2^)

**Table:**

	*U*^11^	*U*^22^	*U*^33^	*U*^12^	*U*^13^	*U*^23^
Br1	0.0479 (6)	0.0445 (6)	0.1087 (9)	−0.0044 (4)	−0.0059 (5)	−0.0085 (5)
Br2	0.0317 (5)	0.0595 (7)	0.0885 (8)	0.0053 (3)	−0.0116 (4)	−0.0005 (5)
O1	0.032 (3)	0.051 (3)	0.067 (4)	0.002 (2)	−0.009 (3)	−0.001 (3)
N1	0.040 (4)	0.041 (4)	0.055 (4)	0.014 (3)	0.006 (3)	0.003 (3)
C1	0.022 (4)	0.046 (5)	0.043 (4)	0.004 (3)	0.004 (3)	−0.001 (3)
C2	0.032 (4)	0.043 (4)	0.048 (5)	0.002 (3)	0.002 (3)	−0.004 (4)
C3	0.035 (4)	0.040 (5)	0.055 (5)	0.011 (3)	−0.003 (3)	−0.001 (4)
C4	0.025 (4)	0.046 (5)	0.054 (5)	0.003 (3)	0.002 (3)	−0.001 (4)
C5	0.033 (4)	0.046 (5)	0.040 (4)	−0.004 (3)	0.000 (3)	0.004 (3)
C6	0.038 (4)	0.041 (4)	0.040 (4)	0.007 (3)	0.003 (3)	−0.001 (3)
C7	0.038 (5)	0.052 (5)	0.042 (4)	0.008 (3)	0.003 (3)	−0.003 (4)
C8	0.031 (4)	0.052 (5)	0.074 (6)	0.017 (4)	0.004 (4)	0.013 (4)

### Geometric parameters (Å, º)

**Table:**

Br1—C2	1.902 (7)	C3—H3A	0.9300
Br2—C4	1.913 (8)	C4—C5	1.381 (10)
O1—C1	1.328 (9)	C5—C6	1.399 (10)
O1—H1	0.8184	C5—H5A	0.9300
N1—C7	1.274 (10)	C6—C7	1.455 (10)
N1—C8	1.474 (9)	C7—H7A	0.9300
C1—C6	1.396 (10)	C8—C8^i^	1.515 (15)
C1—C2	1.401 (10)	C8—H8A	0.9700
C2—C3	1.372 (10)	C8—H8B	0.9700
C3—C4	1.359 (10)		
			
C1—O1—H1	105.2	C4—C5—H5A	120.7
C7—N1—C8	118.1 (7)	C6—C5—H5A	120.7
O1—C1—C6	123.0 (6)	C1—C6—C5	120.3 (7)
O1—C1—C2	119.4 (6)	C1—C6—C7	121.4 (7)
C6—C1—C2	117.6 (7)	C5—C6—C7	118.3 (7)
C3—C2—C1	122.5 (7)	N1—C7—C6	119.3 (7)
C3—C2—Br1	119.3 (5)	N1—C7—H7A	120.4
C1—C2—Br1	118.1 (6)	C6—C7—H7A	120.4
C4—C3—C2	118.1 (7)	N1—C8—C8^i^	109.0 (8)
C4—C3—H3A	120.9	N1—C8—H8A	109.9
C2—C3—H3A	120.9	C8^i^—C8—H8A	109.9
C3—C4—C5	122.7 (7)	N1—C8—H8B	109.9
C3—C4—Br2	119.7 (5)	C8^i^—C8—H8B	109.9
C5—C4—Br2	117.5 (6)	H8A—C8—H8B	108.3
C4—C5—C6	118.7 (7)		
			
O1—C1—C2—C3	179.0 (7)	O1—C1—C6—C5	−179.2 (7)
C6—C1—C2—C3	−3.2 (12)	C2—C1—C6—C5	3.1 (11)
O1—C1—C2—Br1	−0.6 (10)	O1—C1—C6—C7	1.3 (12)
C6—C1—C2—Br1	177.2 (5)	C2—C1—C6—C7	−176.4 (7)
C1—C2—C3—C4	1.5 (12)	C4—C5—C6—C1	−1.3 (11)
Br1—C2—C3—C4	−179.0 (6)	C4—C5—C6—C7	178.2 (7)
C2—C3—C4—C5	0.4 (13)	C8—N1—C7—C6	180.0 (7)
C2—C3—C4—Br2	−179.6 (6)	C1—C6—C7—N1	0.1 (12)
C3—C4—C5—C6	−0.5 (12)	C5—C6—C7—N1	−179.5 (7)
Br2—C4—C5—C6	179.5 (6)	C7—N1—C8—C8^i^	121.0 (10)

Symmetry code: (i) −*x*+1, −*y*, −*z*+1.

### Hydrogen-bond geometry (Å, º)

**Table:**

*D*—H···*A*	*D*—H	H···*A*	*D*···*A*	*D*—H···*A*
O1—H1···N1	0.82	1.83	2.573 (7)	151
